# Supplementary material for: Empirical methods for controlling false positives and estimating confidence in ChIP-Seq peaks
Source: BMC Bioinformatics. 2008 Dec 5;9:523. doi: 10.1186/1471-2105-9-523 (PMC2628906; doi:10.1186/1471-2105-9-523)
Supplement: Additional File 1 — A variety of html documents from the USeq web site detailing the available applications, their best usage, output file type descriptions, command line menus, etc. [file 1471-2105-9-523-S1.zip › USeqUserGuides/JavaDoc.zip!/JavaDoc/trans/roc/package-summary.html]

trans.roc


|  |  |  |
| --- | --- | --- |
| **Overview** \| **Package** \| Class \| **Use** \| **Tree** \| **Deprecated** \| **Index** \| **Help** | |  |
| **PREV PACKAGE**   **NEXT PACKAGE** | **FRAMES**    **NO FRAMES**     **All Classes** |  |


---

## Package trans.roc

For manipulating the BAC dmel spike in data and generating ROC curves.

**See:**
  
          **Description**

| **Class Summary** | |
| --- | --- |
| **Bin** | Score collector for Sgr apps. |
| **ControlGene** |  |
| **ExonScoreExtractor** | Extracts scores from xxx.bar files for particular groups of exons. |
| **ExtractNonOverlappingWindows** | Parses out non overlapping `RocWindow`s. |
| **ExtractSaveSgrScores** | Application for extracting and saving the scores from a .sgr file as a serialized float[]. |
| **ExtractScoresForRoc** | Assignes values from an sgr file to a collection of `RocWindow`s. |
| **Gr** | Gr line container. |
| **GrComparator** |  |
| **MethodTester** | Application for scoring '.sgr' files for positives and negatives in relation to known (spike in) data. |
| **ParsePatternedWindows** | Finds RocWindows upstream and withing the neighborhood of the ATG start site of a patterned gene list. |
| **ParseSgrsForParticularRegions** | Returns oligo values that overlap particular regions. |
| **ParseSgrsForParticularScores** | Returns oligo values that overlap particular regions. |
| **Positive** | Container for a positive BAC used by the Sgr scripts. |
| **PositiveComparator** |  |
| **PWMScoreWindows** | Scores windows for hits to a transcription factor PWM, for `RocWindow` filtering. |
| **RocWindow** | For holding information about a region for ROC analysis. |
| **RocWindowScanner** | Scans a window array over a range of thresholds and intersects with a list of positives to generate the false positive rate, true positive rate, and false discovery rates. |
| **ScoreParsedBars** | Returns values that overlap particular regions, can generate p-values associated with random back ground model |
| **Sgr** | Sgr line container. |
| **SgrBacCounter** | Application for scoring '.sgr' files for positives and negatives in relation to the BAC spike in data. |

## Package trans.roc Description

For manipulating the BAC dmel spike in data and generating ROC curves.

---


|  |  |  |
| --- | --- | --- |
| **Overview** \| **Package** \| Class \| **Use** \| **Tree** \| **Deprecated** \| **Index** \| **Help** | |  |
| **PREV PACKAGE**   **NEXT PACKAGE** | **FRAMES**    **NO FRAMES**     **All Classes** |  |


---
